# Giant Cell Arteritis Following Dental Procedure

**DOI:** 10.7759/cureus.99419

**Published:** 2025-12-16

**Authors:** Ahmad Jabaiah

**Affiliations:** 1 Internal Medicine, University of California Los Angeles David Geffen School of Medicine, Los Angeles, USA

**Keywords:** giant cell arteritis (gca), large vessel vasculitis and gca, root canal therapy, temporal arteritis (ta), trismus

## Abstract

Giant cell arteritis (GCA), also known as temporal arteritis, is a chronic vasculitis affecting large- and medium-sized arteries in patients over 50 years old. Untreated GCA can cause serious morbidity like irreversible vision loss, aneurysms, or stroke. We present a case of a patient who developed an acute headache and trismus within hours of undergoing a root canal treatment. Because GCA typically presents more gradually, the sudden onset of symptoms raised concern for dental-related complications or infection, contributing to a delay in considering GCA. This case highlights the importance of maintaining a broad differential and recognizing atypical presentations to ensure timely diagnosis and treatment, thereby reducing the risk of long-term morbidity.

## Introduction

Giant cell arteritis (GCA) is a chronic granulomatous vasculitis of the large- and medium-sized arteries that has a predisposition to affect the cranial arteries but can also affect the aorta and its major branches. Inflammation is focused along the internal elastic lamina of the arteries, leading to occlusion of the blood vessel or weakening of the walls, which could lead to the formation of an aneurysm, dissection, or rupture. It is the most common vasculitis in patients of Northern European descent aged 50 years or older, with a prevalence of 52 cases per 100,000 in the United States [1-4].

Common presentations of GCA include headaches, scalp tenderness, jaw claudication, constitutional symptoms (weight loss, fatigue, fever, and night sweats), visual changes, and elevated inflammatory markers. Symptom onset is typically gradual and will vary depending on whether the inflammation is predominantly cranial or extracranial [3]. 

GCA is an important cause of irreversible vision loss. It can affect one or both eyes. The mechanism of vision changes includes arteritic anterior ischemic optic neuropathy (A-AION), central or branch retinal artery occlusion, posterior ischemic optic neuropathy, and cerebral ischemia. A-AION is the most common mechanism of vision loss in GCA caused by occlusion of the posterior ciliary artery, causing ischemia to the optic nerve [5,6].

In 2022, the American College of Rheumatology and the European Alliance of Associations for Rheumatology (EULAR) published classification criteria for GCA. According to these criteria, one of the requirements for diagnosis is being ≥ 50 years of age. Other criteria are to be ascertained from history, physical, and diagnostic tests [5,6]. A positive temporal artery biopsy is the gold standard for diagnosing GCA, and obtaining a color Doppler ultrasound (CDUS) prior to biopsy and early in the treatment course can improve the sensitivity of the biopsy [7,8]. 

Systemic corticosteroids are the mainstay of treatment for GCA. The treatment course will be dictated by the presence or lack of vision loss. The dosage of oral steroids is maintained for 2 to 4 weeks, after which a slow taper may be initiated based on clinical improvement and improvement in the inflammation markers [7]. Tocilizumab is a monoclonal antibody IL-6 receptor antagonist that is FDA-approved for the treatment of GCA and can be considered for initial treatment or as adjunct therapy with corticosteroids [5,9]. 

## Case presentation

A 72-year-old male with a significant past medical history of psoriasis, osteoporosis, and hypertension presented to an outpatient clinic for evaluation of two weeks of bilateral jaw pain, pressure on both sides of his head, and trouble opening his mouth. Symptoms started hours after a left upper tooth root canal procedure. Symptoms initially started with left-sided jaw and head pressure before progressing over the course of a week. The patient’s dentist diagnosed the patient with a dental infection, and he completed one week of penicillin VK with no improvement in symptoms. The patient was concerned he had tetanus, as he worked with sheet metal and did not recall when his last vaccine booster had been. The patient was recommended to receive a tetanus booster at his pharmacy and to return to his dentist for further treatment of dental infection. 

Two weeks later, the patient returned to the clinic with continued symptoms of pain on both sides of his head, trouble opening his mouth, and trouble chewing. The physical exam noted the blood vessels on his temples were tender and bulging. The patient was on amoxicillin for a suspected dental infection from his dentist. 

On exam, the patient was afebrile and hemodynamically stable. His temperature was 36.4℃, pulse was 75 beats/min, blood pressure was 153/85, and respiratory rate was 16 breaths/min. The HEENT exam showed a left upper tooth with an area of erythema on the gum base and no discharge. There was tenderness to palpation over both jaws, especially over his temporomandibular joints (left greater than right). The patient was able to open his jaw to about a 3-finger breadth with discomfort. Based on his history and exam, the patient was recommended to present to the emergency department for expedited work-up and biopsy to evaluate for giant cell arteritis.

Table 1 shows the laboratory results.

**Table 1:** Laboratory results

Test	Result	Normal Range
WBC (White Blood Cell Count)	10.13 (H)	4.16 – 9.95 x10E3/uL
Hemoglobin	12.5 (L)	13.5 – 17.1 g/dL
Platelet Count	272	143 - 398 x10E3/uL
Creatinine	0.87	0.60 – 1.30 mg/dL
ESR (Erythrocyte Sedimentation Rate)	60 (H)	≤ 12 mm/hr
C-reactive protein	5.4 (H)	< 0.8 mg/dL
Hepatitis B surface Antigen	Nonreactive	Nonreactive
Hepatitis C antibody screen	Nonreactive	Nonreactive
Tuberculosis (T-SPOT)	Negative	Negative

Diagnostic tests

CT face with contrast showed no evidence of facial abscess.

Ultrasound duplex bilateral temporal arteries

Wall thickening of the bilateral temporal arteries may support the diagnosis of giant cell arteritis in the appropriate clinical setting.

Pathology of left temporal artery biopsy

Active arteritis with fibrinoid necrosis and fibrosis. Moderate intimal hyperplasia with focal calcification. Sulfated alcian blue and Congo red stains are negative for amyloid deposition. 

**Figure 1:**
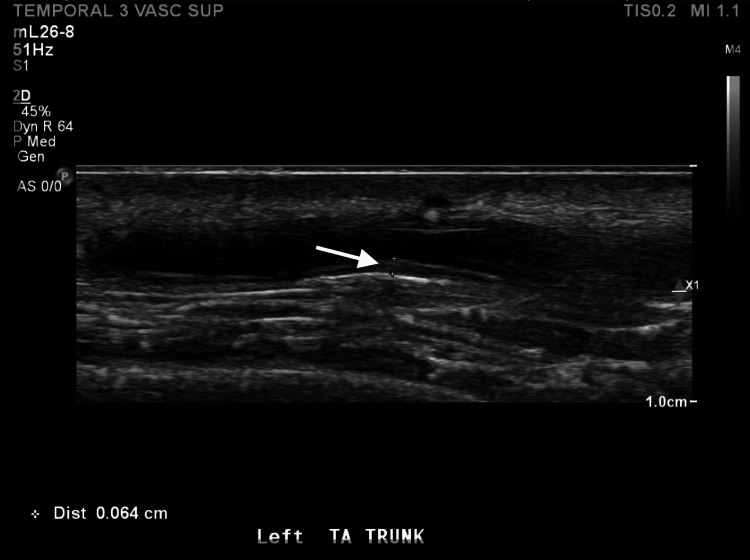
Sagittal view of the left common superficial temporal artery showing intima media thickness of 0.64 mm. Normal cutoff is 0.5 mm.

While hospitalized, a facial CT ruled out dental infection or abscess. A color Doppler ultrasound (CDUS) was suggestive of giant cell arteritis (Figure 1). The patient was started on prednisone 60 mg daily and was evaluated by ophthalmology, who found no evidence of ocular manifestations of GCA. The patient subsequently underwent a temporal artery biopsy performed by oculoplastics and was discharged home on prednisone. Pathology confirmed the diagnosis of GCA, scoring 12 points in the diagnostic criteria (Table 2) [3], and the decision was made to initiate steroid-sparing treatment with intravenous tocilizumab at 6 mg/kg every four weeks due to his osteoporosis and history of lumbar compression fracture. The patient was also placed on PJP prophylaxis with double-strength sulfamethoxazole-trimethoprim every other day and on the proton pump inhibitor (PPI) pantoprazole 40 mg daily for the prevention of gastric ulceration while his prednisone was being tapered. Both PJP prophylaxis and the PPI were discontinued once the prednisone dosage decreased below 20 mg per day.

**Table 2:** 2022 American College of Rheumatology/EULAR classification criteria for giant cell arteritis [3]

Classification Criteria for Giant Cell Arteritis	
Absolute Requirement:	
Age ≥ 50 years at time of diagnosis	
Additional Clinical Criteria:	
Morning Stiffness in Shoulders/Neck	+2
Sudden Visual Loss	+3
Jaw or tongue Claudication	+2
New Temporal Headache	+2
Scalp Tenderness	+2
Absent or diminished pulses, tenderness or hard “cord-like” appearance of temporal arteries.	+2
Diagnostics:	
Maximum ESR ≥ 50 mm/hr or maximum CRP ≥ 10mg/liter prior to initiation of treatment	+3
Positive temporal artery biopsy or halo sign on temporal artery ultrasound	+5
Bilateral axillary involvement (stenosis or aneurysm) noted on imaging	+2
PDG-PET uptake throughout aorta	+2
Sum the scores for 10 items. A score of ≥ 6 points is needed for the classifications of giant cell arteritis	

The patient reported rapid improvement of symptoms, and his inflammatory markers ESR and CRP both normalized a month after initiation of prednisone. The patient reports feeling back to his normal state of health and is off prednisone. He continues monthly intravenous tocilizumab under the direction of rheumatology. 

## Discussion

Our patient's presentation of bilateral headache and jaw claudication in a Caucasian male over the age of 50 with elevated inflammatory markers is typical of GCA. Other symptoms of GCA that were absent in our patient include constitutional symptoms (weight loss, fatigue, fever, and night sweats) and visual changes. Symptoms in this case were sudden, occurring hours after a dental procedure. Symptom onset is typically gradual in GCA and will vary depending on whether the inflammation is predominantly cranial or extracranial [3]. Approximately half of patients will present with concurrent symptoms of polymyalgia rheumatica (PMR), with symptoms of morning stiffness and myalgias in the shoulder and pelvic girdle. The majority of GCA patients will exhibit some cranial manifestation. Large vessel involvement is usually identified upon imaging; however, signs and symptoms from large artery vasculitis that are uncommon include arm or leg claudication, arterial bruits, and asymmetric blood pressure readings [5].

Our patient did not exhibit vision loss. Vision loss in GCA can be sudden and painless and can develop in 35 to 60% of untreated patients. Once vision loss manifests, treatment does not guarantee vision recovery, with only 15 to 34% of patients treated having had some improvement in their vision loss. Visual changes can present initially as transient visual loss (amaurosis fugax) resembling a curtain effect that spontaneously resolves after a few minutes. This is caused by reduced perfusion of the optic nerve, retina, or choroid. Untreated cases will progress to permanent vision loss within one week [5,6]. 

Important laboratory and diagnostic tests include checking the inflammatory markers, erythrocyte sedimentation rate (ESR), and C-reactive protein (CRP), which are usually elevated in GCA, keeping in mind that ESR’s upper limit of normal changes with age and between males and females. The gold standard for the diagnosis of GCA remains a temporal artery biopsy (TAB) [5]. Performing a CDUS can suffice for the diagnosis of GCA if the findings show a “halo sign,” a hypoechoic ring around the artery lumen, indicating inflammation-induced edema in the temporal artery. CDUS was noted to have sensitivity and specificity ranging from 55 to 100% and 78 to 100%, respectively. Other imaging modalities like MRI with angiography, 18FDG-PET CT, and CT with angiography can be used to evaluate for vasculitis. MRI angiography exhibits sensitivity and specificity comparable to those of CDUS, being 68 to 89% sensitive and 73 to 97% specific, while 18F-FDG-PET has a reduced specificity of around 66% [7]. CDUS can also be used to aid in locating a suitable TAB site, considering that the segmented nature of GCA reduces the rates of false-negative biopsies. Diagnostic CDUS should be performed before initiation of treatment or within a week of starting treatment, as the positive findings rapidly resolve after initiation of treatment, with time ranging between 3 and 22 days [7,8]. Performing a CDUS prior to TAB improves the sensitivity, as the biopsy is directed to the temporal artery segment with the positive findings [8].

Management of GCA will depend on the presence of visual symptoms. Like our patient who was without vision loss, these cases are initiated on high-dose oral prednisone of around 1 mg/kg daily, with average daily doses between 60 and 80 mg. Patients with vision loss are treated with pulsed intravenous methylprednisolone for three days, followed by oral prednisone. The dosage of oral prednisone is maintained for 2 to 4 weeks, after which a slow taper may be initiated based on clinical improvement and improvement in the inflammation markers ESR and CRP. After the 2- to 4-week mark, prednisone is tapered by 10 mg every two weeks until a dose of 20 mg daily is reached. If the patient remains in remission, further prednisone tapering is done by 1 to 2.5 mg every two weeks until discontinuation [7]. Treatment with high doses of systemic corticosteroids has many adverse events, including infection, hypertension, hyperglycemia, gastric ulcers, osteoporosis, and mood changes. Screening for latent TB is recommended, as well as prophylactic treatment for Pneumocystis jirovecii pneumonia (PJP) until the prednisone dosage has been reduced to less than 20 mg a day. Due to the many adverse effects of long-term high-dose corticosteroids, steroid-sparing medications are used to reduce the iatrogenic harm of steroids. Tocilizumab, a monoclonal antibody IL-6 receptor antagonist, can be considered for initial treatment or as adjunct therapy with corticosteroids [5,9].

GCA relapse after a period of remission can occur and is frequently seen. Up to 45% of patients on corticosteroid monotherapy will exhibit elevation in ESR and CRP along with their clinical manifestations. Those on tocilizumab monotherapy or concomitantly with steroids will have the clinical manifestations without the elevation in ESR and CRP, making relapse more challenging to confirm. Relapse tends to occur most frequently in the two years after diagnosis and rarely occurs after the fifth year. Treatment with corticosteroid monotherapy and rapid titration of therapy to less than 12 months is associated with relapse. Relapses can be classified as major or minor based on severity. A major relapse includes signs and symptoms of ischemia, like vision changes, jaw or limb claudication, or stroke. Major relapse is treated with the reinstating of high-dose corticosteroids as in the initial treatment. Minor relapses include symptoms like headaches, isolated PMR, or constitutional symptoms and are treated by increasing the corticosteroid dose to the previous effective dosage or by increasing it by 5 to 15 mg/day. Both major and minor relapses should prompt initiation of adjunctive therapy with the addition of tocilizumab if on corticosteroid monotherapy or, if on both corticosteroid and tocilizumab therapy, modification of tocilizumab dosage or frequency. Those who fail tocilizumab can be started on methotrexate. For patients on corticosteroid monotherapy, the tapering from 60 mg of prednisone to less than 20 mg daily is done within the first three months, then a slow taper to 5 mg daily at the one-year mark. For those on concomitant therapy with tocilizumab, corticosteroids are tapered within the first six months while tocilizumab is continued. With about half of patients treated with tocilizumab developing relapse after one year of discontinuation of the medication, the duration of tocilizumab will depend on clinical outcome and history of relapse [1].

Follow-up is recommended every 1 to 3 months in the first year and then every 3 to 6 months thereafter. Because aortic aneurysms are a late complication, patients should be monitored after discontinuation of therapy with imaging, although modality and frequency will depend on the patient and their history [1].

## Conclusions

Giant cell arteritis is a serious vasculitis of medium- and large-sized arteries. Although incidence increases with age and northern European ancestry, no direct correlation was found between recent dental procedures, surgery, or trauma and disease onset. GCA’s clinical presentation varies based on the vascular territory involved, with cranial symptoms being most common. Prompt diagnosis is critical, especially to prevent irreversible complications such as blindness. High-dose corticosteroids remain the cornerstone of treatment. Steroid-sparing agents like tocilizumab are also an option for initial treatment and as adjunct therapy. Despite appropriate therapy, relapse is common, particularly within the first two years, highlighting the importance of individualized treatment plans and long-term monitoring.
